# Traumatic peripheral nerve injuries: a classification proposal

**DOI:** 10.1186/s10195-023-00695-6

**Published:** 2023-05-10

**Authors:** Andrea Lavorato, Gelsomina Aruta, Raffaele De Marco, Pietro Zeppa, Paolo Titolo, Michele Rosario Colonna, Mariarosaria Galeano, Alfio Luca Costa, Francesca Vincitorio, Diego Garbossa, Bruno Battiston

**Affiliations:** 1 Neurosurgery Unit, Igea Hospital, via Marcona 69, 20129 Milan, Italy; 2 Department of Neurosciences “Rita Levi Montalcini”, Neurosurgery Unit, University of Turin, Turin, Italy; 3 Traumatology–Reconstructive Microsurgery, Department of Orthopedics and Traumatology, CTO Hospital, Turin, Italy; 4 Department Human Pathology, University of Messina, Viale Della Libertà 395, 98121 Messina, Italy; 5 Department of Biological Imaging and Morphology, University of Messina, Messina, Italy; 6 Clinic of Plastic Surgery, Department of Neurosciences, University of Padua, Padua, Italy

**Keywords:** Peripheral nerve, Injuries, Classification, Prognostic factors

## Abstract

**Background:**

Peripheral nerve injuries (PNIs) include several conditions in which one or more peripheral nerves are damaged. Trauma is one of the most common causes of PNIs and young people are particularly affected. They have a significant impact on patients’ quality of life and on the healthcare system, while timing and type of surgical treatment are of the utmost importance to guarantee the most favorable functional recovery. To date, several different classifications of PNIs have been proposed, most of them focusing on just one or few aspects of these complex conditions, such as type of injury, anatomic situation, or prognostic factors. Current classifications do not enable us to have a complete view of this pathology, which includes diagnosis, treatment choice, and possible outcomes. This fragmentation sometimes leads to an ambiguous definition of PNIs and the impossibility of exchanging crucial information between different physicians and healthcare structures, which can create confusion in the choice of therapeutic strategies and timing of surgery.

**Materials:**

The authors retrospectively analyzed a group of 24 patients treated in their center and applied a new classification for PNI injuries. They chose (a) five injury-related factors, namely nerve involved, lesion site, nerve type (whether motor, sensory or mixed), surrounding tissues (whether soft tissues were involved or not), and lesion type—whether partial/in continuity or complete. An alphanumeric code was applied to each of these classes, and (b) four prognostic codes, related to age, timing, techniques, and comorbidities.

**Results:**

An alphanumeric code was produced, similar to that used in the AO classification of fractures.

**Conclusions:**

The authors propose this novel classification for PNIs, with the main advantage to allow physicians to easily understand the characteristics of nerve lesions, severity, possibility of spontaneous recovery, onset of early complications, need for surgical treatment, and the best surgical approach.

Level of evidence: according to the Oxford 2011 level of evidence, level 2.

## Introduction

Traumatic peripheral nerve injuries (PNIs) include several conditions producing damage of one or more peripheral nerves, together with possible loss of motor or sensory functions. Trauma is one of the most common causes of PNI in the general population and the most common in young people, with an incidence estimated between 1.46% and 2.8%, especially in the upper extremities [1–3]. Nerve injuries that occur during specific sports account for less than 0.5%, but recent studies suggest a higher rate in the USA [4].

In the upper limbs, the radial nerve is the most frequently involved, followed by the ulnar nerve, and the median nerve. In the lower limbs, the sciatic nerve is the most frequently damaged, followed by the peroneal nerve [2, 5]. Among the forces responsible for injuries, traction, transection, radiation, compression, thermal, and electrical forces must be mentioned, as they produce the same pathophysiological effects, including demyelination and Wallerian degeneration [6].

When approaching PNI, it is crucial to consider the significant impact they have on patients’ quality of life and on healthcare systems. PNIs, indeed, affect mostly young and economically active people, who suffer from various degrees of disability and from neuropathic pain [7, 8], which is a chronic and especially challenging condition for patients with PNI, leading to a reduction in autonomy during activities of daily living (ADLs) and precluding job opportunities and forcing people to adopt chronic use of painkillers and other drugs [9–11]. Moreover, orthoses, largely used to reduce pain, may be very uncomfortable and aesthetically unpleasant for patients with PNI [12, 13].

The most favorable recovery can be achieved through early diagnosis, correct timing, and type of surgery [14], but can be affected by several factors such as age [15], gender [11], comorbidities, type and level of injury, and the presence of concomitant injuries [16]. With regard to time, in our experience, a delay in identification and treatment of PNIs is often seen, especially in road accident victims. Late referral to specialized centers or concomitant other life-threatening conditions, such as head or thoracic traumas, produce a crucial delay in PNI diagnosis and treatment, and irreversibly affect the outcome [17–21].

Correct diagnosis is paramount for surgical planning, as bone, vessel and muscle, and multiple nerve injuries may also be associated [2, 22, 23], and a single surgery may not be enough to restore satisfactory function; in some cases, multiple surgeries are required [23–25]. Moreover, revision surgery is not infrequent, especially for patients with delayed diagnosis [26–28]. Identifying precise clinical and surgical data through the application of a univocal language could produce more accurate exchanges between hospitals and surgeons and allow surgeons to better plan revision surgeries.

In this paper, we describe a novel anatomo-topographical and prognostic classification, in which two distinct types of alphanumeric code can be applied to all PNIs to guide physicians in the decision process. We introduced this classification in our center to verify its feasibility, and it has been adopted among physiatrists, physiotherapists, neurologists, and surgeons in a multidisciplinary setting.

## Materials and methods

We chose the following five injury-related factors to create the alphanumeric code:

- **Nerve involved**: axillary or circumflex (A),   suprascapular (Su), long thoracic (LT), thoracodorsal (TD), musculocutaneous (Mu), median (Me), radial (R), ulnar (U), common digital (CD), sciatic (Sc), peroneal (P), tibial (T)
- **Lesion site**: 1 shoulder/pelvis, 2 arm/thigh, 3 elbow/knee, 4 forearm/leg, 5 wrist–hand/ankle–foot
- **Nerve type**: 1 motor, 2 sensory, 3 mixed
- **Surrounding tissues**: open (O), closed (C)
- **Lesion type**: partial/in continuity (P), complete (C) (modifiers: 1 clean, 2 crushed, 3 loss of tissue);

Regarding the prognostic alphanumeric code, we considered the following four factors:

- **Age**: aged (A—more than 60), young (B)
- **Timing**: 1 immediate, 2 delayed, 3 secondary
- **Technique**: 1 suture, 2 graft ≤ 6 cm, 3 graft > 6 cm.
- **Comorbidities** (smoking, diabetes, etc.): yes (Y), no (N)

Each alphanumeric code can unambiguously be associated with a management strategy and specific treatment options. The prognostic alphanumeric code, calculated on patient- and treatment-related factors, such as age, comorbidities, and surgical technique, guides the physician in predicting time of recovery and prognosis.

A complete description of our classification is summarized in Table 1.

**Table 1:**
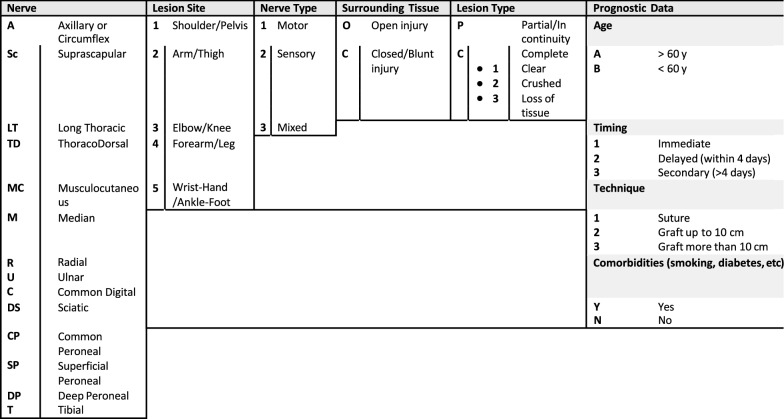
Peripheral nerve injury code description

Extensive application of the alphanumeric code was carried out in 24 patients treated in our center (Table 2), also reporting the surgical treatment we chose for each patient.

**Table 2:** Table of patients

Patient no.	Sex (M:F = 20:4)	Age at surgery (years) (average, 37 years)	Diagnosis	Mechanism of injury	PNI code	Treatment
1	M	15	Right external popliteal nerve injury	Deep clear-cut injury at proximal peroneal epiphysis	SP33OC/B11	Neurorraphy
2	M	28		Subamputation of III, IV, and V fingers with cutaneous injury to the volar portion of MCP joint	CD52OC/B11	Extensors and flexors tenorraphy and neurorraphy
3	M	43	Posterior circumflex nerve and its first branches (superficial and deep) injury	Complete lesion at the quadrangular space or quadrilateral space of Volpeau	C13CC/B22	Axillary nerve neurolysis, subsequent nerve transfer of axillary nerve with tricipital branch and sural autograft of 25 mm, and open-surgery for rotator cuff repair
4	M	68	Median nerve and sensitive branch of radial nerve injuries	Inveterate lesion with 6 mm gap between stumps at median nerve and 38 mm gap between stumps of the sensitive branch of radial nerve	M43CC R42CC/A22	Reconstruction with sural autografts for median and sensory radial branch
5	M	23	Sensitive palmar branch of median nerve injury	Forearm open wound chronically progressed with 25 mm gap between stumps	M52CC/B22	Neurorraphy of sensory palmar branch of median nerve and posterior interosseous nerve grafting of 25 mm
6	F	42	Common digital nerve injury	Iatrogenic lesion, result of right hand aponeurectomy with evidence of neuroma-in-continuity in digital branch for IV finger, and 25 mm gap between stumps at bifurcation of common digital nerve on the V finger	CD52CC/B22	Reconstruction with sural autografts
7	M	44	Median and ulnar nerves injury	Two level amputation at radiocarpal and scaphotrapeziotrapezoid joints	M43OC U43OC/B11	Neurorraphy
8	M	15	Zone III ulnar nerve injury	Complete inveterate lesion just distal to ulnar collateral ligament with 40 mm gap between stumps	U43CC/B22	Reconstruction with five sural autografts (40 mm)
9	M	33	Radial Nerve Injury	Inveterate lesion of the proximal third of the upper arm with 60 mm gap between stumps	R23CC/B22	Reconstruction with five dural autografts
10	F	30	Common digital nerve injury	Complete amputation of III finger of the left hand	CD52OC/B11	Neurorraphy
11	M	29	Ulnar nerve injury	Complete lesion at middle third of the forearm with 50 mm gap between stumps	U33CC/B22	Reconstruction with four sural autografts (50 mm)
12	M	56	Ulnar nerve injury	Complete inveterate lesion 4 cm proximal to Guyon’s canal with 25 mm gap between stumps	U23CC/B22	Reconstruction with neurotube
13	M	25	Sciatic nerve injury	Inveterate lesion at proximal third of thigh with 80 mm gap between stumps	S23CC/B22	Neurolysis and reconstruction with five sural autografts (80 mm)
14	M	18	Palmar digital nerve injury	Complete interphalangeal joint (IPJ) amputation	CD52OC/B11	Neurorraphy
15	M	46	Median, ulnar, and radial nerves injuries	Complete distal third forearm amputation	M43OC U43OC R42OC/B11	Neurorraphy
16	M	54	Radial nerve injury	Nerve lesion at the level of the wrist with 14 mm gap between stumps	R52CC/B22	Reconstruction with muscle–vein-combined grafts
17	M	22	Common digital nerve injury	I finger amputation at metacarpophalangeal joint (MPJ)	CD52OC/B11	Neurorraphy
18	M	69	Ulnar nerve injury	Inveterate lesion at proximal third of the forearm with 40 mm gap between stumps	U43CC/A22	Reconstruction with five sural autografts
19	M	46	Radial nerve injury	Inveterate posterior cord of brachial plexus (BP) lesion, distal to axillary nerve origin with 60 mm gap between stumps	R1CC/B22	Reconstruction with 4 sural autografts
20	M	52	Common digital nerve injury	Open wound with loss of substance of the III finger with 12 mm gap between stumps	CD52CC/B22	Reconstruction with muscle–vein-combined grafts
21	F	31	Sciatic nerve injury	Partial injury of the left sciatic nerve	S23CP/B22	Neurorraphy
22	M	36	Peroneal nerve Injury	Inveterate lesion of superficial peroneal nerve at fibular head with 120 mm gap between stumps	SP33CC/B23	Reconstruction with two sural autografts (240 mm)
23	F	52	Median and musculocutaneous nerve injury	Inveterate lesion with 150 mm and 80 mm gap between stumps, respectively	M1 3CC MC13CC/B23	Reconstruction with two sural autografts
24	M	12	Ulnar nerve injury	Inveterate lesion at elbow level with 40 mm gap between stumps	U33CP/B22	Reconstruction with two sural autografts (40 mm)

## Results

Surgery was decided because of the above-mentioned anatomo-topographical and prognostic factors, and a multidisciplinary team was able to retrospectively propose the same therapeutic strategies thanks to the clear definition of the lesion.

Every alphanumeric code could unambiguously be associated with a management strategy and specific treatment options. The prognostic alphanumeric code, calculated on patient- and treatment-related factors, such as age, comorbidities, and surgical technique, guided the physician in predicting time of recovery and prognosis.

## Discussion

As for spine injuries [29] and fractures to the appendicular skeleton [30], our classification with its alphanumerical system moves a step forward in describing PNI through the improvement in inter- and intraobserver reliability, helping to address both surgeons’ decision process and prognosis prediction.

Historically, in the context of PNI, the first attempt to classify nerve injury was made by Seddon [31], considering the correlation between pathological evidence and outcome. Since nerve regeneration was seen only in class I and II injuries corresponding to neuroapraxia and axonotmesis, respectively, a surgical option was justified in the early period in class III injuries (neurotmesis) where the surrounding connective tissue of the nerve was disrupted. Following Seddon’s classification, Sunderland created a five-point grading system of PNI severity in ascending order, giving a more detailed description of surrounding connective tissue damage [32]. Connective tissue is spared in grade II injury, while increased involvement of the connective layers surrounding the nerve fibers, namely endoneurium, perineurium, and epineurium, defines a progressively worse injury requiring surgical intervention. Mackinnon [33] added a grade VI to Sunderland’s classification, to include PNIs with mixed pattern and, consequently, to better reflect clinical practice. There is no reference to other elements in the cited classifications that may influence the outcome, such as the traumatic mechanism of the nerve lesion.

Among general classifications, Millesi et al. [34] proposed an analysis to guide surgeons during neurolysis procedures. The authors focused on the site of fibrosis that could occur at different levels after a traumatic injury. They explained different pathogenetic mechanisms through which scar tissue in different layers could impair the nerve and may affect the clinical picture. Specifically, the authors defined 4 types of fibrosis correlating to Sunderland’s grade, requiring specific neurolytic procedures: fibrosis of the epifascicular epineurium (type A) needs epifascicular epineurotomy, when the scar tissue involves the interfascicular epineurium (type B) it is necessary to remove the epineurium layer performing an epineurectomy and, to free deeper layers, it is often associated with partial interfascicular epineurectomy, and in type C fibrosis the endoneurium is involved and the presence of neurolysis aids in the making of a diagnosis. In the original article, a type D was reported corresponding to loss of fascicular pattern observed during neurolysis. This classification represents a first attempt to categorize the nerve fibrosis that could occur after a trauma—or after surgery—and to guide surgeons during surgical decision making. However, considering just the “effect” of the lesion without the underlying “mechanism” and level of injury, the information is partial and incomplete for correct surgical management.

We could say that Seddon, Sunderland, MacKinnon, and Millesi described the injury per se as far as it concerns the nerve trunk.

Other recent studies attempted a classification of single nerve injuries as far as it concerns the nerve’s regional anatomy, with their surgical treatment proposals. Ghoraba et al. [35] have recently proposed an algorithm to assess ulnar injuries considering four anatomical zones: distal to the proximal hiatus of Guyon’s canal (zone I), from the proximal hiatus of Guyon’s canal to the proximal border of the pronator quadratus (zone II), from the proximal border of the pronator quadratus to the first motor branch of the ulnar nerve (zone III), and proximal to the first motor branch of the ulnar nerve (zone IV). They managed injuries to zone I and II, primarily, with neurorrhaphy or with an autologous nerve graft, and more proximal lesions (zone III and IV) with anterior transposition. Although the study shows good results, the proposed classification still remains limited to a single anatomical compartment.

In the case of brachial plexus injuries (BPIs), Millesi et al. [36] identified four anatomical sites of injury, namely (I) supraganglionic/preganglionic, (II) infraganglionic/postganglionic, (III) trunk, and (IV) cord. Indeed, this simplification for surgical management obtained good correlation with outcome. Improvement and diffusion of imaging techniques brought Yang et al. [37] to characterize five types of BPIs with the aid of magnetic resonance imaging (MRI). MRI was used to locate BPIs in relation to the preganglionic nerve root and postganglionic spinal nerve, to guide the surgical strategy and to formulate a provisional prognosis. Unfortunately, despite specificity, none of these classifications takes into account certain preoperative factors that can affect functional outcomes after treatment, and few classifications prioritize the management and prognosis of specific nerve injuries.

There are many works that have emphasized that loss of nerve substance, local ischemia, and extensive tissue damage [5, 38, 39], as well as other patient-related factors such as age [15] or smoking [40], could all be possible causes of impaired nerve regeneration. Other elements that have been investigated as influencing factors to the nerve repairing process include the mechanisms of injury [41–43], with evidence of better sensory and motor recovery for clean-cut injuries compared with crush and avulsion injuries. Additional factors, such as operative delay (despite the heterogeneity of results in literature [20, 32, 44, 45]), the level of injury, and operative timing, were analyzed showing better recovery for distal compared with proximal injuries [46–48], while there is no consensus in the literature on the role of operative delay in the outcome of PNIs [11, 38].

Given all these limits, a first attempt proposing a more complete classification was made by Goubier et al. [49]: they considered several preoperative factors, such as type of injury, delay of motor nerve repair, level of motor nerve injury, age of patient, perioperative smoking, and management in microsurgical unit, which have been demonstrated to have some impact on the outcome, to predict the final prognosis of peripheral nerve lesions. However, because of the absence of validation of this scale, it is of limited use in surgeons’ decision-making process for therapeutic strategies and surgical timing.

Our classification can improve the lack of information of the previously published attempts, as it can describe the type of lesion in a complete and exhaustive way, particularly concerning the localization and the extension of injuries and the surrounding tissues.

In fact, injuries to long nerves, such as median, radial, ulnar, and sciatic nerves, running through different limb segments, and also in the case of multiple levels of injury, can appropriately be described (our class is “lesion site”), and the type of injuries and the conditions of the surrounding tissues (our class defines whether close or open) are also clearly ascribable as well.

The following practical examples can show how the classification can be applied extensively and give complete information.

The alphanumeric code can in fact be applied to multiple-level lesions by sequentially describing the different levels of the lesion after repeating the code for the nerve. For example, R42CC/A22 R23CC/A22 is a code representing a lesion of the radial nerve in an elderly subject on two different levels in the limb. In this way, the classification applies to many complex traumas with lesions of the same nerve on several levels.

As another example, R23C3B2Y is the alphanumeric prognostic code associated with an injury of the radial nerve (R) at the arm level (2), where the nerve is mixed (3), the injury is now presenting as closed (C) with loss of substance (3), in a young patient (B), with delayed treatment (2), treated with a graft > 6 cm (2), and the patient presents with comorbidities (Y). As in this case, even limited information about the type of surgical reconstruction and anamnestic data could be enough to help nerve surgeons and clinicians in predicting a prognosis for a particular PNI case.

Regarding inveterate lesions, applying this code, for example, a complete inveterate sciatic nerve lesion, localized at proximal third of the thigh, with a 8 cm gap, is associated with the alphanumeric code Sc23CC3 (Sc sciatic nerve, 2 thigh, 3 lesion of a mixed nerve, C surrounding tissue closed, C3 complete with loss of tissue). The clear definition of the lesion leads to adopting the treatment of neurolysis and reconstruction with nerve (sural) grafts.

For inveterate PNIs, a unique clinical assessment might be helpful in a multidisciplinary environment [50]. Physiatrists could draft a rehabilitation program according to the “code” of nerve injury, finalize it after clinical evaluation, and train physiotherapists accordingly. Electrophysiologists could set up their equipment and detect a nerve lesion more critically, if the clinical question is well described: a clear dialogue between specialists leads to more efficient management of patients over time.

For acute trauma cases with PNI, remote consultation is rarely applicable, and time is life changing. Early ultrasound imaging and electrophysiologic tests are operator-dependent, whereas MRI may suffer from some limitations for acute nerve injuries [51]. Clinical evaluation in these cases is one of the most important elements to obtain a correct diagnosis and proper clinical management. An “identity (ID) code” for nerve lesions provides clear, immediate, and unambiguous information about a specific clinical condition and the related preferable surgical treatment, when applicable. If shared, this classification might easily permit smart dialogue between hospitals, especially when patients need to be treated in a center that is different from the one where a PNI diagnosis was originally made.

Outcome evaluation always depends on correct categorization of nerve lesions [52]. The more a univocal classification is shared among healthcare centers, the lower the data bias registered during follow-up evaluations. Results in different patients—and also in a single patient over time—would be directly comparable, leading to more accurate clinical management of patients and a higher standard of care. In clinical practice, nerve injuries are treated in specialized centers, which are often different from the center where a PNI diagnosis is made. An all-embracing classification for these particular traumatic lesions allows clinicians to speak the same language between different healthcare centers. Even a non-experienced physician could classify PNI cases correctly to provide complete and clear information to the reference center.

Our classification might indirectly suggest the first surgical approach, by giving physicians unambiguous information about nerve lesions, nature of injury, and connective tissue involvement. The PNI code leads to more precise, careful and realistic surgical planning, which translates into more complete, straight-forward information for patients and families. With the proposal of an alphanumeric classification, we attempted to create an all-encompassing classification of PNI that can help physicians and healthcare workers to efficiently manage these injuries in daily practice. Even the most complex, multilevel injuries, such as those involving all the plexuses and the facial nerve, can be described by this method. The anatomical site of injury in each nerve course is also included in the classification, which helps in single nerve exploration. Indeed, this advantage should also be kept in mind as we consider that single classifications of nerve injury have been proposed for the radial nerve [53], and for facial nerve injury [54] based on their anatomy. Those classifications focus on the single nerve injury and fit the lesion well, but our classification also may include this “single” classification. Regarding lesion type, with the help of this classification, we can predict outcome and adapt to evolving clinical/instrumental findings, which are crucial in neuropraxia. Here the condition may change over time, and a clinical and instrumental reappraisal is needed.

Moreover, it is often very difficult to code, even via a detailed physical examination, NCV/EMG, and image study. All surgeons of the peripheral nerves know that a definite diagnosis needs intraoperative exploration. We believe that in these special cases, the classification and the coding variations may be useful, also as a retrospective tool documenting any changes in a complete way. The final purpose is to spread knowledge and awareness for nerve lesion cases among healthcare personnel and to encourage communication and data exchange between different medical centers, to guarantee the best possible treatment and care for patients with PNI.

## Conclusions

Like so many other pathological entities, PNIs need a correct clinical assessment and categorization to supply all the necessary information for the precise understanding and management of these patients. Our PNI-code represents a clear, all-embracing classification, able to ensure that physicians are speaking the same language when approaching an acute or chronic nerve injury. We believe that sharing basic information can assure good clinical practice, even for complex cases or with non-experienced workers, maintaining the same high standards of care between different medical centers.

Our practical attempt to use this classification for 24 patients showed its feasibility in clinical practice. We strongly believe that a clinical and anamnestic PNI-code will not only be useful in defining single cases or case series, but could also represent a valid tool to suggest indications and define prognosis. The real situation might be further described by finding out whether single PNI-code subcategorizations correlate with different clinical outcomes. A good lesion definition may guarantee correct data analysis, thereby reducing bias.

The weakness of our study lies in the retrospective nature of our data, together with the limited and heterogeneous number of cases taken into consideration, which does not allow us to relate the application of our classification to the definition of treatments and prognosis in this study.

The correlation between the classification of PNIs and prognosis, therefore, goes beyond the scope of this study, which is limited to the definition of a descriptive classification that is made to be as complete as possible, and also includes those in the literature that are recognized as the main prognostic factors.

However, the utility of our alphanumeric classification might grow enormously with its spread and acceptance in the scientific community, especially in the matter of revision and second surgeries, where its utility can be easily appreciated even with a small number of cases.

On the other hand, coding using multiple alphabets and numbers is difficult to memorize as it needs to cover the entire clinical scenario in its current format, but we are dealing with a complex matter, and complexity cannot be reduced if one must have complete information. Emergency department and all physicians involved in the treatment of peripheral nerve injuries may use our classification chart, just as burn centers use the Lund and Browder chart.

Thus, new, larger studies are needed to improve the outcome prediction reliability and accuracy of this classification in management and treatment recommendations.

## Data Availability

The datasets generated and/or analyzed during the current study are not publicly available due to Italian National Law on Privacy, but are available from the corresponding author on reasonable request.

## References

[1] Kouyoumdjian J, Graç C, Ferreira VM (2017). Peripheral nerve injuries: a retrospective survey of 1124 cases. Neurol India.

[2] Noble J, Munro CA, Prasad VSSV, Midha R (1998). Analysis of upper and lower extremity peripheral nerve injuries in a population of patients with multiple injuries. J Trauma Acute Care Surg.

[3] Taylor CA, Braza D, Rice JB, Dillingham T (2008). The incidence of peripheral nerve injury in extremity trauma. Am J Phys Med Rehabilit.

[4] Olivo R, Tsao B (2017). Peripheral nerve injuries in sport. Neurol Clin.

[5] Robinson LR (2000). Traumatic injury to peripheral nerves. Muscle Nerve.

[6] Ferrante MA (2018). The assessment and management of peripheral nerve trauma. Curr Treat Options Neurol.

[7] Ciaramitaro P, Mondelli M, Logullo F (2010). Traumatic peripheral nerve injuries: epidemiological findings, neuropathic pain and quality of life in 158 patients. J Periph Nervous Syst.

[8] Miclescu A, Straatmann A, Gkatziani P (2019). Chronic neuropathic pain after traumatic peripheral nerve injuries in the upper extremity: prevalence, demographic and surgical determinants, impact on health and on pain medication. Scand J Pain.

[9] Colloca L, Ludman T, Bouhassira D (2017). Neuropathic pain. Nat Rev Dis Primers.

[10] Girach A, Julian TH, Varrassi G (2019). Quality of life in painful peripheral neuropathies: a systematic review. Pain Res Manag.

[11] Liu X, Zhu J, He B (2014). Factors predicting sensory and motor recovery after the repair of upper limb peripheral nerve injuries. Neural Regen Res.

[12] Bettoni E, Ferriero G, Bakhsh H (2016). A systematic review of questionnaires to assess patient satisfaction with limb orthoses. Prosthet Orthot Int.

[13] Magnusson L, Ghosh R, Jensen KR (2019). Quality of life of prosthetic and orthotic users in South India: a cross-sectional study. Health Qual Life Outcomes.

[14] Dahlin LB, Wiberg M (2017). Nerve injuries of the upper extremity and hand. EFORT Open Reviews.

[15] Stratton JA, Eaton S, Rosin NL (2020). Macrophages and associated ligands in the aged injured nerve: a defective dynamic that contributes to reduced axonal regrowth. Front Aging Neurosci.

[16] Galanakos SP, Zoubos AB, Ignatiadis I (2011). Repair of complete nerve lacerations at the forearm: an outcome study using Rosén-Lundborg protocol—repair of complete nerve lacerations. Microsurgery.

[17] Battiston B, Titolo P, Ciclamini D, Panero B (2017). Peripheral nerve defects. Hand Clin.

[18] Brunelli G, Brunelli F (1990). Strategy and timing of peripheral nerve surgery. Neurosurg Rev.

[19] Dahlin LB (2013). The role of timing in nerve reconstruction. Int Rev Neurobiol.

[20] Jivan S, Kumar N, Wiberg M, Kay S (2009). The influence of pre-surgical delay on functional outcome after reconstruction of brachial plexus injuries. J Plast Reconstr Aesth Surg.

[21] Jonsson S, Wiberg R, McGrath AM (2013). Effect of delayed peripheral nerve repair on nerve regeneration, Schwann cell function and target muscle recovery. PLoS ONE.

[22] Huckhagel T, Nüchtern J, Regelsberger J, Lefering R (2018). TraumaRegister DGU Nerve injury in severe trauma with upper extremity involvement: evaluation of 49,382 patients from the TraumaRegister DGU® between 2002 and 2015. Scand J Trauma Resusc Emerg Med.

[23] Narakas AO (1985). The treatment of brachial plexus injuries. Int Orth.

[24] Limthongthang R, Bachoura A, Songcharoen P, Osterman AL (2013). Adult brachial plexus injury: evaluation and management. Orthop Clin North Am.

[25] Songcharoen P, Mahaisavariya B, Chotigavanich C (1996). Spinal accessory neurotization for restoration of elbow flexion in avulsion injuries of the brachial plexus. J Hand Surg.

[26] Cain JD, Di Nucci K (2009). Revisional peripheral nerve surgery. Clin Podiatr Med Surg.

[27] Kaiser R, Ullas G, Havránek P (2017). Current concepts in peripheral nerve injury repair. Acta Chir Plast.

[28] Vora AM, Schon LC (2004). Revision peripheral nerve surgery. Foot Ankle Clin.

[29] Vaccaro AR, Oner C, Kepler CK (2013). AOSpine thoracolumbar spine injury classification system: fracture description, neurological status, and key modifiers. Spine.

[30] Müller ME, Koch P, Nazarian S, Müller ME, Nazarian S, Koch P, Schatzker J (1990). Principles of the classification of fractures. The comprehensive classification of fractures of long bones.

[31] Seddon HJ (1943). Three types of nerve injury. Brain.

[32] Sunderland S (1951). A classification of peripheral nerve injuries producing loss of function. Brain.

[33] Mackinnon SE (1989). New directions in peripheral nerve surgery. Ann Plast Surg.

[34] Millesi H, Rath T, Reihsner R, Zoch G (1993). Microsurgical neurolysis: its anatomical and physiological basis and its classification. Microsurg.

[35] Ghoraba SM, Mahmoud WH, Elsergany MA (2019). Ulnar nerve injuries (Sunderland Grade V): a simplified classification system and treatment algorithm. Plast Reconstr Surg Global Open.

[36] Millesi H (1988). Brachial plexus injuries: nerve grafting. Clin Orthop Relat Res.

[37] Yang J, Qin B, Fu G (2013). Modified pathological classification of brachial plexus root injury and its MR imaging characteristics. J Reconstr Microsurg.

[38] Wang E, Inaba K, Byerly S (2017). Optimal timing for repair of peripheral nerve injuries. J Trauma Acute Care Surg.

[39] Wang ML, Rivlin M, Graham JG, Beredjklian PK (2019). Peripheral nerve injury, scarring, and recovery. Connect Tissue Res.

[40] Rodriguez-Fontan F, Reeves B, Tuaño K, Colakoglu S, D’Agostino L, Banegas R (2020). Tobacco use and neurogenesis: a theoretical review of pathophysiological mechanism affecting the outcome of peripheral nerve regeneration. J Orthop.

[41] Gelberman RH, Urbaniak JR, Bright DS, Levin S (1978). Digital sensibility following replantation. J Hand Surg.

[42] Jaeger SH, Tsai T-M, Kleinert HE (1981). Upper extremity replantation in children. Orth Clin North Am.

[43] Shergill G, Bonney G, Munshi P, Birch R (2001). The radial and posterior interosseous nerves: results of 260 repairs. J Bone Joint Surg.

[44] Fu SY, Gordon T (1995). Contributing factors to poor functional recovery after delayed nerve repair: prolonged denervation. J Neurosci.

[45] Khalifa H, Belkheyar Z, Diverrez J-P, Oberlin C (2012). Results of 24 nerve repairs at more than one year post-injury. Chir Main.

[46] Hundepool CA, Ultee J, Nijhuis THJ (2015). Prognostic factors for outcome after median, ulnar, and combined median–ulnar nerve injuries: a prospective study. J Plast Reconstr Aesth Surg.

[47] Ruijs ACJ, Jaquet J-B, Kalmijn S, Giele H, Hovius SER (2005). Median and ulnar nerve injuries: a meta-analysis of predictors of motor and sensory recovery after modern microsurgical nerve repair. Plast Reconstr Surg.

[48] Sakellarides H (1962). A follow-up study of 172 peripheral nerve injuries in the upper extremity in civilians. J Bone Joint Surg Am.

[49] Goubier J-N, Teboul F, Tubbs RS, Rizk EB, Shoja M, Loukas M, Barbaro N, Spinner RJ (2015). Grading of Nerve Injuries. Nerves and nerve injuries: vol 2: pain, treatment, injury, disease and future directions.

[50] Lavorato A, Raimondo S, Boido M (2021). Mesenchymal stem cell treatment perspectives in peripheral nerve regeneration: systematic review. Int J Molec Sci.

[51] Martín-Noguerol T, Montesinos P, Barousse R, Barousse A (2021). RadioGraphics update: functional MR neurography in evaluation of peripheral nerve trauma and postsurgical assessment. Radiographics.

[52] Roganović Z (1998). Factors influencing the outcome of nerve repair. Vojnosanit Pregl.

[53] Pan CH, Chuang DC, Rodríguez-Lorenzo A (2010). Outcomes of nerve reconstruction for radial nerve injuries based on the level of injury in 244 operative cases. J Hand Surg Eur.

[54] Amer TA, El Kholy MS, Khalaf AA, Rifki AM (2021). Amer’s classification of territories of facial nerve injury in early cases and strategies for the management of different territories. J Plast Reconstr Aesthet Surg.

